# P-490. Association of Vascular and Inflammatory Markers with Neurocognitive Test Performance among Ugandan Youth with Perinatally Acquired HIV on Dolutegravir

**DOI:** 10.1093/ofid/ofaf695.705

**Published:** 2026-01-11

**Authors:** Caroline Carlson, Shan Sun, Christine Karungi, Joy Louise Gumikiriza-Onoria, Courtney Kirsch, Angel Nanteza, Christopher Ferraris, Namal Liyanage, Nicholas Funderburg, Victor Musiime, Reuben N Robbins, Sahera Dirajlal-Fargo

**Affiliations:** Northwestern University Feinberg School of Medicine, Chicago, Illinois; Ann & Robert H. Lurie Children’s Hospital of Chicago, Chicago, Illinois; Joint Clinical research Centre, Kampala, Wakiso, Uganda; Makerere University College of Health Sciences, Kampala, Kampala, Uganda; New York State Psychiatric Institute and Columbia University, Metuchen, New Jersey; Butabika National Referral Mental Hospital, Kampala, Kampala, Uganda; New York State Psychiatric Institute, New York, New York; The Ohio State University, COLUMBUS, Ohio; The Ohio State University, COLUMBUS, Ohio; Joint Clinical Research Center, Kampala, Uganda, Kampala, Kampala, Uganda; Columbia University and New York State Psychiatric Institute, New York, New York; Northwestern University, Chicago, Illinois

## Abstract

**Background:**

The association between neuroinflammation and neurocognition in youth with perinatally acquired HIV (YPHIV) has been documented. However, mechanisms underlying neurocognition in YPHIV on contemporary ART (cART) in Sub-Saharan Africa remain unclear. This study examined associations of vascular, inflammatory, and gut markers with neurocognition in YPHIV virally suppressed on dolutegravir vs. youth without HIV (YWoH).

**Figure:**
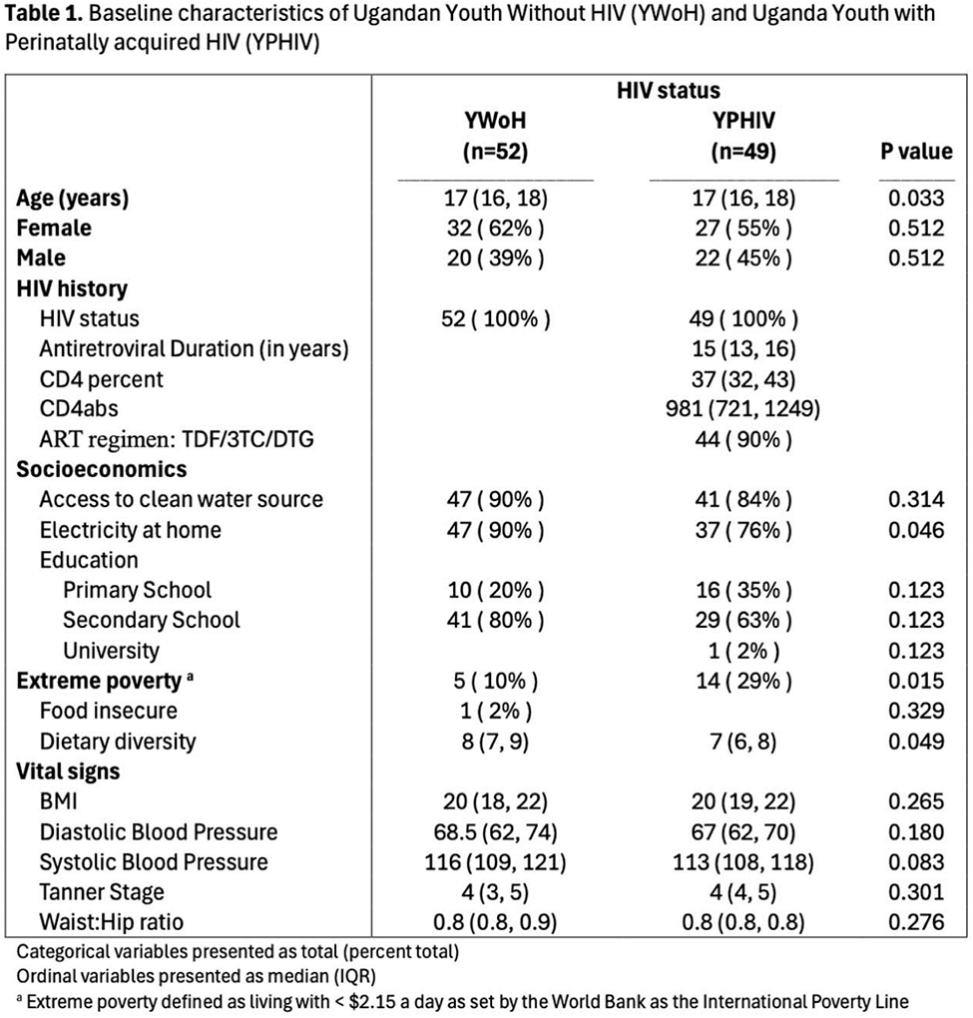

**Figure:**
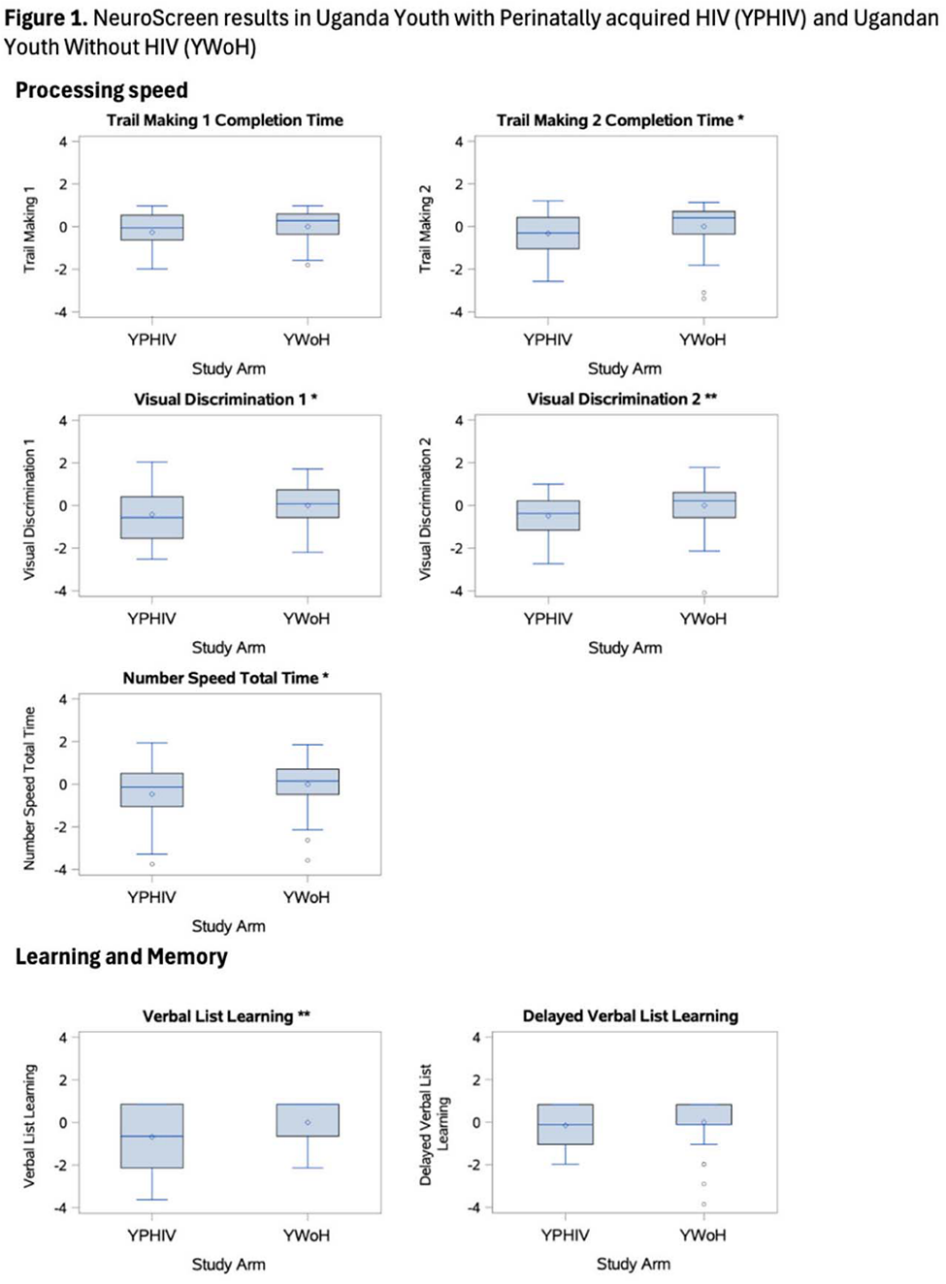

**Methods:**

A cross-sectional study of 101 youth in Kampala, Uganda was conducted (52 YWoH and 49 YPHIV). Neurocognition was measured with NeuroScreen (NS), a tablet-based test battery adapted for Uganda. YWoH based individual test z-scores and a global z-score were calculated. Plasma markers of inflammation, chemokines, gut microbial translocation, and vascular markers were measured using ELISA and Legendplex. Wilcoxon rank sum test compared neurocognitive measures. Spearman correlations assessed associations of neurocognition with biomarkers. General linear regression models assessed the association between neurocognition and biomarkers after adjusting for demographic, socioeconomic, and HIV factors.

**Figure:**
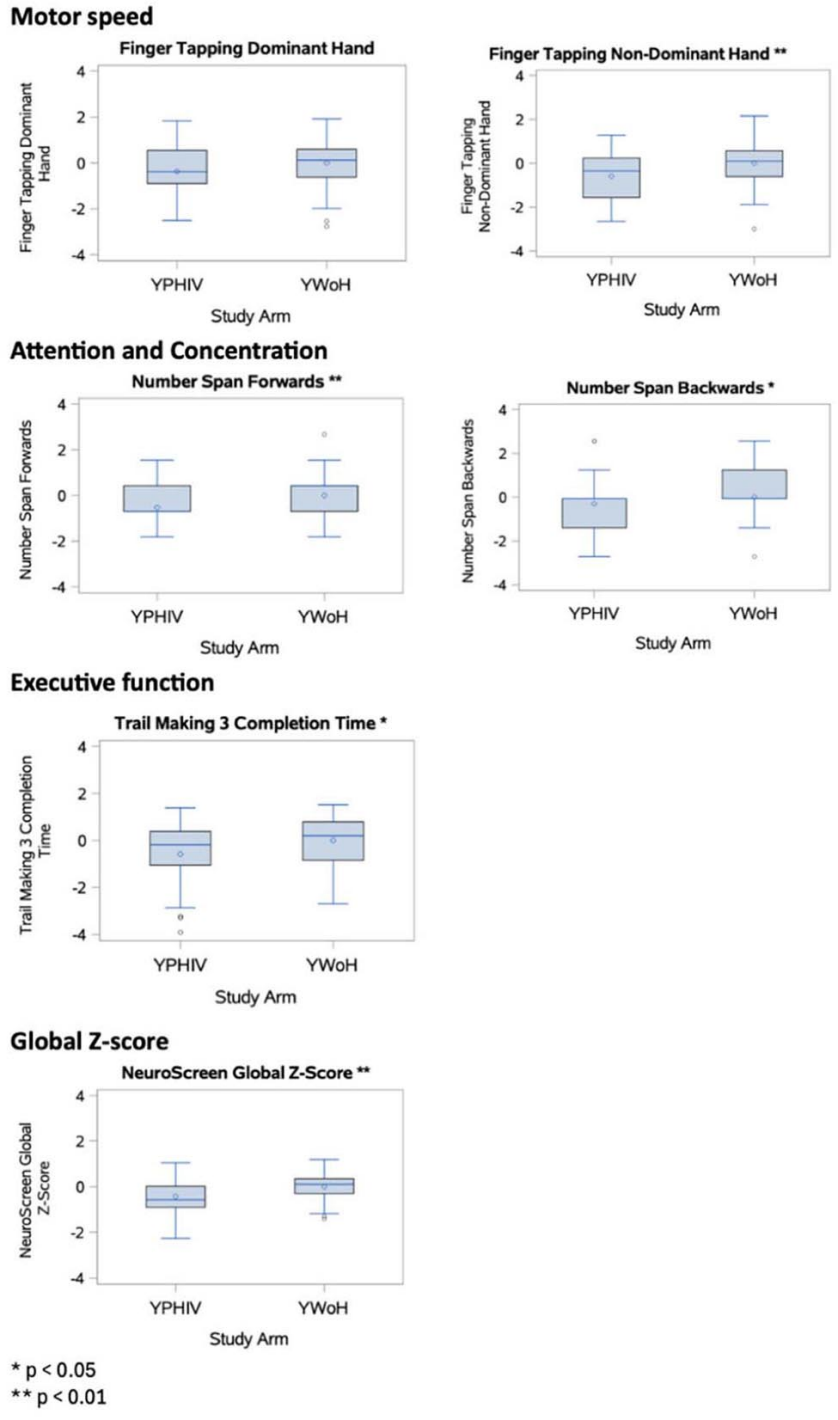

**Figure:**
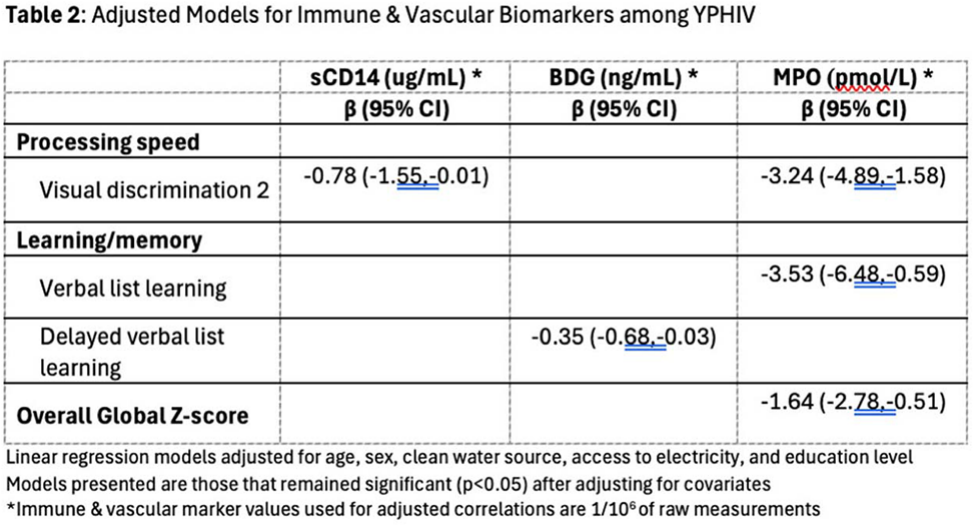

**Results:**

The median [IQR] age was 16.90 years [15.11, 17.08] and 42% were females (Table 1). Compared with YWoH, YPHIV participants performed worse in all neurocognitive domains and had lower global z-scores (p ≤ 0.038, Figure 1). Gut integrity marker IFAB and chemokines MIP3 and RANTES were greater in YPHIV compared to YWoH (p ≤ 0.024). After adjustment, the vascular marker myeloperoxidase (MPO) was associated with worse processing speed, learning/memory, and global z-scores in YPHIV, while MPO was only associated with worse attention/concentration in YWoH. Among YPHIV only, monocyte activation marker sCD14 and fungal translocation marker BDG were associated with worse processing speed and learning/memory, respectively (Table 2).

**Conclusion:**

Despite viral suppression on cART regimens, Ugandan YPHIV performed poorly on neurocognitive tests compared to YWoH. Immune markers associated with mortality and cardiovascular disease in HIV, such as MPO, sCD14 and BDG, were associated with worse neurocognitive performance in Ugandan youth with HIV, suggesting vascular inflammation and gut barrier dysfunction processes that persist with cART therapy.

**Disclosures:**

Caroline Carlson, BA, Elekta Foundation: Rayos Contra Cancer employee salary supported by the Elekta Foundation to build educational programs Nicholas Funderburg, PhD, Gilead: Grant/Research Support

